# The impact of a rapid molecular identification test on positive blood cultures from critically ill with bacteremia: A pre-post intervention study

**DOI:** 10.1371/journal.pone.0223122

**Published:** 2019-09-26

**Authors:** Alexia Verroken, Noémie Despas, Hector Rodriguez-Villalobos, Pierre-François Laterre

**Affiliations:** 1 Department of Microbiology, Cliniques Universitaires Saint-Luc – Université Catholique de Louvain, Brussels, Belgium; 2 Intensive Care Department, Cliniques Universitaires Saint-Luc – Université Catholique de Louvain, Brussels, Belgium; National Yang-Ming University, TAIWAN

## Abstract

**Objectives:**

Bloodstream infections in critically ill require a speeded-up microbiological diagnosis to improve clinical outcomes. In this pre-post intervention study, we evaluated how a molecular identification test directly performed on positive blood cultures of critically ill improves patient’s therapeutic management.

**Methods:**

All adult patients staying at the intensive care unit (ICU) at the time of positive blood culture detection were study-eligible. In the 8-month pre-intervention period (P0), standard positive blood culture management was performed. In the 10-month intervention period (P1), a BioFire^®^ FilmArray^®^ blood culture identification (FA-BCID) test (bioMérieux) was additionally performed 24/7 at detection. The evaluated clinical outcome was time to optimal antimicrobial treatment of the bloodstream infection. FA-BCID microbiological test performances were also analysed.

**Results:**

163 positive blood culture episodes were allocated to P0 and 166 to P1. After the withdrawal of episodes in accordance with defined exclusion criteria, outcome analysis was performed on 110 bloodstream infections both in P0 and P1. Time to optimal antimicrobial treatment in P0 was 14h41 compared to 4h39 in P1. FA-BCID test results led to a treatment adjustment in 35/110 (31.8%) P1 episodes including 26 where the adjustment was the optimal antimicrobial treatment. FA-BCID testing identified 96.2% of the on-panel microorganisms thereby covering 85.2% of our ICU-strain epidemiology. Time to identification with FA-BCID testing was calculated at 1h35. Resistance detection was in complete concordance with routine results. Considering 150 FA-BCID tests were initially performed in P1, 4,3 tests were required to have 1 test leading to an improved therapeutic outcome.

**Conclusions:**

FA-BCID testing drastically reduced time to optimal antimicrobial treatment in critically ill with bloodstream infections.

## Introduction

A bloodstream infection (BSI) is caused by the presence of an infective microorganism in the blood of the patient and is associated with major morbidity and mortality rates. Its suspicion generates a cascade of diagnostic and therapeutic measures including blood culture sampling [1]. Rapid detection of a pathogen in the blood cultures is crucial to improve patient’s outcome. Molecular diagnostic approaches starting from positive blood cultures stand out for their short turn-around-time to results but depreciate through their high costs and lack of information on the strains’ susceptibility profiles [2]. Considering the clinical impact of molecular BSI-identification tools, several authors demonstrated a decrease of time to optimal antibiotherapy, a reduced length of hospital-stay and a reduced mortality rate compared to phenotypic identification methods [3–6]. A recent review further concluded molecular rapid diagnostic testing should be considered part of standard of care in patients with BSIs [7]. Yet complementary studies are necessary to identify specific patient-groups for whom molecular testing results could majorly improve clinical outcome. The intensive care unit (ICU) copes with a BSI prevalence of 7.8% and an associated mortality rate of 30–40% [8–9]. Calculated as the third most common infection, ICU-BSIs moreover lead to extended length of stay and major hospital costs [10–11]. It seems therefore obvious that the ICU population could be a valuable candidate to benefit from rapid molecular BSI diagnosis. A largely evaluated molecular tool is the BioFire^®^ FilmArray^®^ blood culture identification (FA-BCID) panel (BioFire Diagnostics, Inc., Salt Lake City, UT, USA, a bioMérieux Company) designed to identify 24 microorganisms and 3 antimicrobial resistance genes (*mecA*, *vanA/B* and *bla*_*KPC*_) in 1hour and 5minutes directly from blood of positive culture bottles [3,5,7]. To our knowledge, this test was never distinctively evaluated on an ICU-population. In this pre-post intervention study, we measured the clinical impact of the FA-BCID test in an ICU-setting with a restrictive antimicrobial policy. We compared microbiological and clinical outcomes of the FA-BCID process performed 24/7 with an already highly optimized positive blood-culture laboratory management process.

## Materials and methods

### Study design and setting

The study was a pre-post intervention study conducted at the Cliniques universitaires Saint-Luc, a tertiary Belgian hospital, with a 22-bed medical-surgery ICU. All patients (≥18 years) with a positive blood culture bottle and remaining in the ICU at the time of positivity detection were enrolled. Exclusion criteria were 1) patients dying between blood culture sampling and positivity detection 2) patients with blood cultures detected positive within 6 hours after incubation 3) patients on palliative care. Also excluded retrospectively were all patients with positive-detected blood cultures but negative subcultures (= false positive blood cultures).

During the pre-intervention period (P0), standard positive blood culture management was performed including Gram stain, matrix-assisted laser desorption ionization time-of-flight mass spectrometry (MALDI-TOF MS) identification and antimicrobial susceptibility testing (AST). Blood cultures were processed according the time frame of positivity detection. This standard procedure was exhaustively described as “the modified workflow” in a previous publication [12]. Briefly blood culture bottles detected between 8 AM and 3 PM had an immediate Gram stain; MALDI-TOF MS identification was available by 5 PM the same day and AST results were available the following day. Bottles detected positive between 3 PM and 11 PM had an immediate Gram stain but were subcultured for MALDI-TOF MS identification performed on the following day; AST results were made available 2 days later. Finally bottles detected positive between 11 PM and 8 AM were managed in the following time frame. Gram stain results were immediately communicated by phone while identification and AST results were transferred upon availability into the patients’ computerized medical records.

During the intervention period (P1), routine positive blood culture management was completed with the FA-BCID test performed 24/7 on the first positive blood culture bottle of each episode within the hour following BACTEC FX alarm signal for growth-detection. An identification result (and a resistance gene result where applicable) was immediately communicated by phone to the ICU physician allowing a prompt antimicrobial adjustment in accordance with the local ICU restrictive antimicrobial stewardship guidelines. A “no-organism detected” FA-BCID test result was not communicated.

The duration of the study periods (in months) was defined in real time based on the inclusion of a minimum of 150 positive blood culture episodes both in P0 and P1 hereby allowing a valid study population comparison. Demographic and clinical characteristics of the patients as well as positive blood culture data were compared for all episodes included in P0 versus P1 to evaluate the similarity of the 2 study populations.

### Outcomes

Evaluated clinical outcome was median time to patient’s administration of the optimal antimicrobial BSI treatment (OAT). The OAT was defined as the final and best-targeted drug prescribed by the ICU physician guided by the local ICU antimicrobial guidelines. Treatment modifications were labeled as either the initiation of an antimicrobial treatment, a de-escalation of the empirical treatment or a spectrum broadening of the empirical treatment. Median time to OAT for each period was calculated on BSIs (not on contaminations) excluding episodes of patients dying within 24 hours after blood culture positivity detection. The empirical treatment was defined as the antimicrobial drug administered before availability of any laboratory results. In accordance with the ICU restrictive antimicrobial policy, an empirical antibiotic was exclusively given to patients with a suspected sepsis. Depending on the recent medical history of the patient, the suspected infectious source and previous antimicrobial therapy, the administered treatment was either a narrow-spectrum antibiotic (i.e. cefuroxime for a community-acquired pneumonia) either a broad-spectrum antibiotic (i.e. piperacillin/tazobactam for a hospital-acquired intra-abdominal infection). All data on BSI and antibiotic management were ultimately reviewed by an adjudication committee composed of an intensive care praticioner and a microbiologist. This study aims to demonstrate a reduced time to OAT in P1 BSIs compared with P0 BSIs.

In parallel, identification and resistance detection performances of the FA-BCID panel were evaluated and median time to positive blood culture strain identification was measured in comparison with standard laboratory management approaches. All time measurements started at the time blood culture bottles were detected positive by the incubators.

### Data handling and statistical analyses

Microbiological data of the positive blood culture episodes were recorded from the laboratory information system and patients’ medical records were reviewed for collection of demographic and clinical characteristics as well as antibiotic treatment data. Classification of positive blood culture episodes into BSI or contamination was defined according to the US Centers for Disease Control and Prevention/National Healthcare Safety Network definitions of bloodstream infection events [13].

Comparisons among the 2 groups were performed using the Mann-Whitney U test for continuous nonparametric variables and Fisher exact test or chi^2^ test for categorical variables; *P* values <0.05 were considered statistically significant. The program GraphPad Prism 6.0e (San Diego, California, United States) was used to perform statistical analysis.

## Results

**Fig 1:**
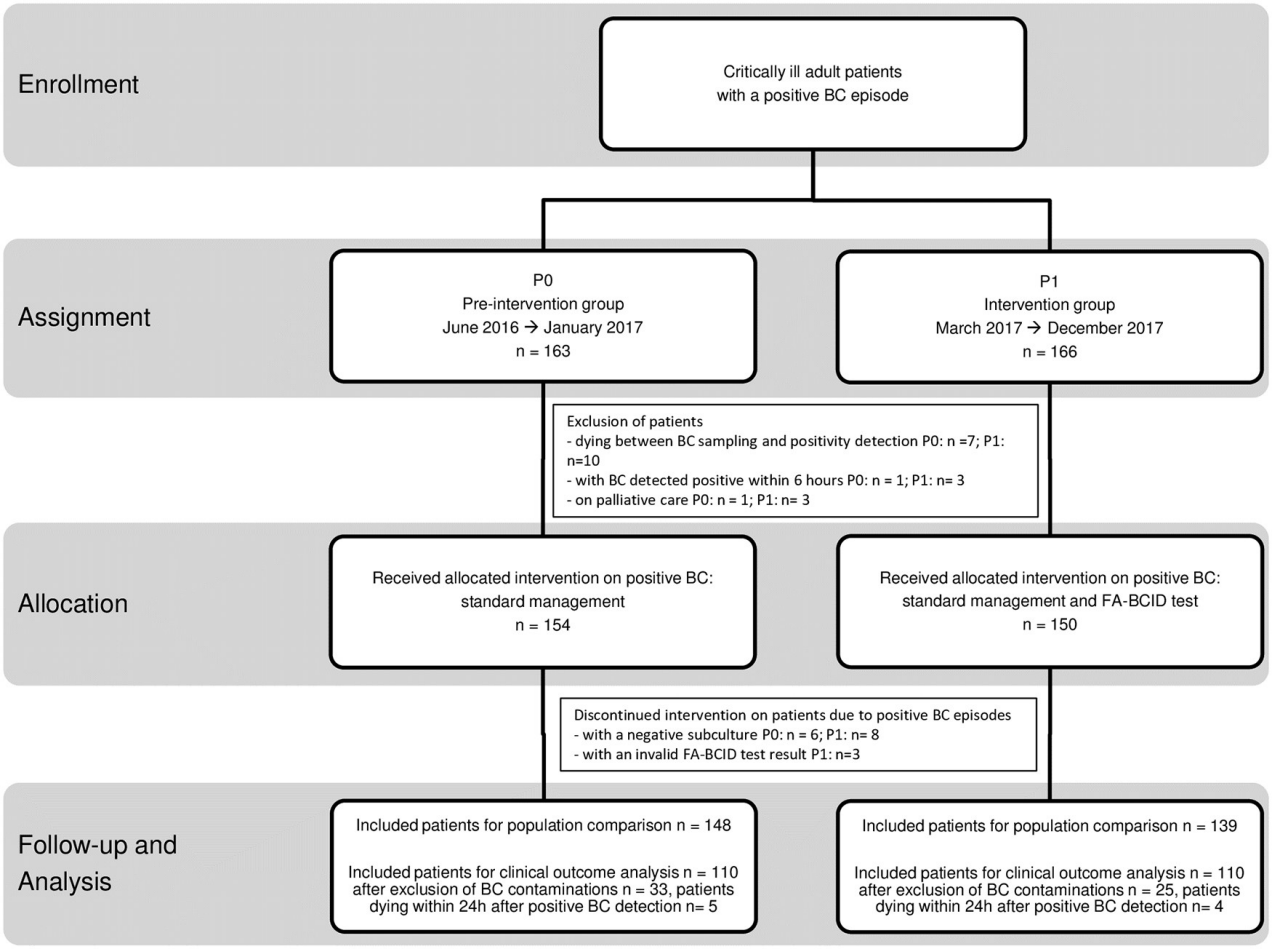
Study inclusion process of critically ill patients with a positive blood culture episode among pre-intervention and intervention groups. Abbreviations: BC, blood culture; FA-BCID, FilmArray blood culture identification; P0: pre-intervention period; P1, intervention period.

The study inclusion process of the critically ill patients with a positive blood culture episode is presented in Fig 1. A total of 163 patients were assigned to P0 going on from June 2016 to January 2017 and 166 patients were assigned to P1 going on from March 2017 to December 2017. Subsequently after the exclusion of 9 patients in P0 and 16 patients in P1 in accordance with defined criteria, 154 and 150 patients received the allocated intervention in respectively P0 (standard positive blood cultures management) and P1 (standard positive blood cultures management and FA-BCID test). However intervention was discontinued on 6 patients in P0 with positive blood culture detection yet negative subcultures. In P1, 8 patients were identically excluded as well as 3 patients with invalid FA-BCID test results. Ultimately 148 and 139 patients with positive blood culture episodes were analyzed in respectively P0 and P1. Clinical characteristics and positive blood culture data of all patients were compared across the 2 study periods and detailed in Table 1. Despite statistical differences in a few analysed comorbidities, the 2 patient groups were comparable in relation to their severity scores. Regarding the distribution of the positive blood culture episodes, contaminations and BSIs accounted for respectively 22.3% (33/148) and 77.7% (115/148) in P0 and 18% (25/138) and 82% (114/138) in P1. Proportions of Gram-positive, Gram-negative and yeast strains were analogous in the 2 study periods. Main BSI sources were the gastro-intestinal tract, the urinary tract and the respiratory tract in both study periods. Among all included patients with a BSI, 80.9% (93/115) presented a sepsis syndrome in P0 and 81.6% (93/114) in P1. 30-day mortality rate was respectively calculated at 36.5% (42/115) in P0 and 33.3% (38/114) in P1. Finally, P0 and P1 blood culture episodes were equally distributed into the three time frames of positivity detection.

**Table 1:** Clinical characteristics and positive blood culture data of patients included in the pre-intervention and intervention periods.

	P0 (n = 148)	P1 (n = 139)	*P* value
Patient characteristics			
Age, years, mean ± SD	63.9 ± 15.2	59.8 ± 16.4	0.04
Male sex	102 (68.9)	90 (64.7)	0.53
APACHE II score, mean ± SD	23.2 ± 10.5	21.7 ± 7.9	0.39
SOFA score, mean ± SD	8.8 ± 4.7	8.8 ± 3.6	0.45
Comorbidities			
Diabetes	47 (31.8)	41 (29.5)	0.70
Active hematological malignancy	19 (12.8)	8 (5.8)	0.04
Active solid malignancy	26 (17.6)	29 (20.9)	0.55
Cardiovascular disorder	86 (58.1)	60 (43.2)	0.01
Chronic lung disease	42 (28.4)	18 (12.9)	0.01
Solid organ transplant	10 (6.8)	18 (12.9)	0.11
Neutrophil count <500/μL	5 (3.4)	7 (5)	0.56
Mechanical ventilation	73 (49.3)	61 (43.9)	0.41
Microbiology			
Gram-positive bacteria	82 (51.3)	80 (53.7)	0.73
Gram-negative bacteria	76 (47.5)	66 (44.3)	0.64
Yeast	2 (1.3)	3 (2)	0.67
Contamination	33 (22.3)	25 (18)	0.30
Bloodstream infections			
Bloodstream infection	115 (77.7)	114 (82)	0.30
source			
gastro-intestinal tract	37 (32.2)	41 (36)	0.58
urinary tract	17 (14.8)	13 (11.4)	0.56
respiratory tract	15 (13)	11 (9.6)	0.53
catheter-related	9 (7.8)	10 (8.8)	0.82
other	12 (10.4)	17 (14.9)	0.33
unknown	25 (21.7)	22 (19.3)	0.74
sepsis	93 (80.9)	93(81.6)	1
septic choc	76 (66.1)	73 (64)	0.78
mortality rate at 30 days	42 (36.5)	38 (33.3)	0.68
Blood culture positivity detection time-frame			
08h-15h	45 (30.4)	35 (25.2)	0.36
15h-23h	32 (21.6)	39 (28)	0.22
23h-08h	71 (48)	65 (46.7)	0.91

Data are represented as No. (%) unless otherwise specified.

Abbreviations: APACHE II, Acute Physiology and Chronic health Evaluation II; P0, pre-intervention period; P1, intervention period; SOFA, Sequential Organ Failure Assessment.

### FA-BCID microbiological performances

**Fig 2:**
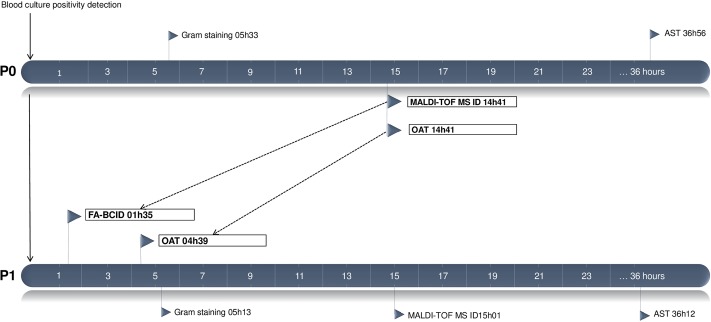
Comparison of median time to microbiological results and time to administration of optimal antimicrobial treatment in critically ill with bloodstream infections included in P0 and P1. Abbreviations: AST, antimicrobial susceptibility testing; FA-BCID, FilmArray blood culture identification; ID, identification; MALDI-TOF MS, matrix-assisted laser desorption ionization time-of-flight; OAT, administration of the optimal antimicrobial treatment; P0, pre-intervention period; P1, intervention period.

From the 139 positive blood culture bottles included in P1, routine blood culture management recovered 149 microorganisms including 75 Gram-positive and 63 Gram-negative strains, 8 anaerobes and 3 yeasts. Detailed MALDI-TOF MS identification results and corresponding FA-BCID results are presented in Table 2. Overall 127 (85.2%) microorganisms were concordantly retrieved with the FA-BCID. 22 (14.8%) strains gave a “no organism detected” FA-BCID result. The latter included 17 off-panel and 5 on-panel strains. Sensitivity of the FA-BCID test in accordance with its on-panel microorganisms was calculated at 96.2% (127/132). Four strains were recovered by FA-BCID but not through routine culture including *Staphylococcus*, *Staphylococcus aureus*, *Streptococcus* and *Klebsiella oxytoca*. The molecular test detected the *mecA* gene in 2/14 S. *aureus* and 25/30 *Staphylococcus* with a sensitivity and specificity of both 100%. Similarly the 7 *vanA/B*-negative *Enterococci* and 59 Gram-negative *BLA*_*KPC*_-negative strains were in concordance with routine AST and no false-positive results were observed. Median time to identification of positive blood culture bottles with the FA-BCID test performed in P1 was reduced to 1h35 in comparison with median time to MALDI-TOF MS identification in P0 and in P1 respectively calculated at 14h41 (*P*<0.05) and 15h01 (*P*<0.05). Additional calculated times to Gram stain results and AST results in P0 and P1 did not show any statistical difference. Microbiological times calculated in the 2 study periods are compared in Fig 2.

**Table 2:** Routine identification results and corresponding FA-BCID results for all strains cultured from the positive blood culture bottles included in the intervention period.

	MALDI-TOF MS ID results (n)		FA-BCID results (n)	
		species ID	genus ID	no organism detected
TOTAL microorganisms	149	81	46	22
Gram-positive bacteria	75	23	41	11
*Staphylococci*				
* Staphylococcus aureus*	14	14	NA	0
* Staphylococcus capitis*	2	NA	1	1
* Staphylococcus epidermidis*	24	NA	24	0
* Staphylococcus haemolyticus*	4	NA	4	0
* Staphylococcus hominis*	2	NA	1	1
*Enterococci*				
* Enterococcus avium*	1	NA	0	1
* Enterococcus casseliflavus*	1	NA	0	1
* Enterococcus faecalis*	6	NA	6	0
* Enterococcus faecium*	1	NA	1	0
*Streptococci*				
Pyogenic group				
* Streptococcus pyogenes*	2	2	NA	0
* Streptococcus dysgalactiae*	1	NA	1	0
Viridans group				
* Streptococcus anginosus* group	2	NA	2	0
* Streptococcus pneumoniae*	6	6	NA	0
* Streptococcus salivarius* group	1	NA	1	0
Other *Streptococci*				
* ****Abiotrophia defectiva***	1	NA	NA	1
Other Gram-positive organisms				
* ****Bacillus cereus***	2	NA	NA	2
* ****Facklamia hominis***	1	NA	NA	1
* ****Gemella morbillorum***	1	NA	NA	1
* ****Lactobacillus rhamnosus***	1	NA	NA	1
* Listeria monocytogenes*	1	1	NA	0
* ****Propionibacterium acnes***	1	NA	NA	1
Gram-negative bacteria	63	55	5	3
Enterobacteriaceae				
* Citrobacter freundii*	1	NA	1^a^	0
* Citrobacter koseri*	1	NA	1^a^	0
* *Enterobacter aerogenes	1	NA	1^a^	0
* Enterobacter cloacae* complex	2	2	NA	0
* Escherichia coli*	38	35	2^b^	1
* Klebsiella oxytoca*	2	2	NA	0
* Klebsiella pneumoniae*	5	5	NA	0
* Serratia marcescens*	2	2	NA	0
Non-fermenters				
* Acinetobacter baumannii*	2	2	NA	0
* Pseudomonas aeruginosa*	6	6	NA	0
* ****Stenotrophomonas maltophilia***	1	NA	NA	1
Other Gram-negative organisms				
* Haemophilus influenzae*	1	1	NA	0
* ***Moraxella nonliquefaciens**	1	NA	NA	1
Anaerobes	8	NA	NA	8
* ****Bacteroides fragilis***	1	NA	NA	1
* ****Bacteroides ovatus***	1	NA	NA	1
* ****Clostridium celerecrescens***	1	NA	NA	1
* ****Clostridium perfringens***	1	NA	NA	1
* ****Clostridium tertium***	1	NA	NA	1
* ****Finegoldia magna***	1	NA	NA	1
* ****Parabacteroides distasonis***	1	NA	NA	1
* ****Parvimonas micra***	1	NA	NA	1
Yeasts	3	3	NA	0
* Candida albicans*	1	1	NA	0
* Candida glabrata*	2	2	NA	0

^a^: Identified to the group level Enterobacteriaceae according to the panel’s abilities;

^b^: Identified to the group level Enterobacteriaceae despite the presence of the species *Escherichia coli* in the panel.

Microorganisms in bold are strains absent from the FA-BCID panel.

Abbreviations: FA-BCID, FilmArray blood culture identification; ID, identification; MALDI-TOF MS, matrix-assisted laser desorption ionization time-of-flight mass spectrometry; NA, not applicable.

### Clinical outcomes

Outcome analysis was performed on 110 patients with bloodstream infections in both periods after exclusion of 5 and 4 patients who died within 24 hours after blood culture positivity detection respectively in P0 and P1.

Median time to administration of the OAT in all BSIs in P1 was 04h39 in comparison with 14h41 in P0 (*P*<0.05) as presented in Fig 2. FA-BCID results led to a treatment modification in 35/110 (31.8%) P1 episodes including 26 where the adjustment was the OAT. The remaining 9 episodes benefitted from additional treatment tailoring following subsequent identification and AST results. Antibiotic switches enabled by FA-BCID results are detailed in Table 3 and were categorized as 21 initiations, 7 de-escalations and 7 spectra broadening of the antimicrobial treatment. Considering the 26 episodes where the FA-BCID result led to the OAT, median time to OAT was 5h24. Among the 9 episodes where FA-BCID results led to a non-OAT switch, 4 of the introduced treatments were too narrow to cover the pathogen(s) requiring further broadening upon availability of additional routine identification and AST results while 5 introduced treatments were too broad and were ultimately reduced. With regards to the time of positivity detection of the blood cultures where FA-BCID results allowed a treatment modification, we observed 7/35 bottles were detected positive between 8 AM and 3 PM, 9/35 in the 3 PM—11 PM time frame and 19/35 in the 11 PM—8 AM time frame.

**Table 3:** P1 blood culture episodes where FA-BCID test results enabled a treatment modification.

	FA-BCID test result	Routine ID result	Treatment switch initiated by FA-BCID test result		
			type	antibiotic	TAT OAT
1	*mecA*-neg *S*. *aureus*	*S*. *aureus*	de-escalation	flucloxacillin	01:10
2	*S*. *pneumoniae*	*S*. *pneumoniae*	initiation	penicillin	01:44
3	*bla*_*KPC*_-neg *E*. *cloacae* complex	*E*. *cloacae* complex	broadening	ciprofloxacin	01:53
4	*mecA*-neg *S*. *aureus*	*S*. *aureus*	initiation	flucloxacillin	02:07
5	*mecA*-pos *S*. *haemolyticus*	*S*. *haemolyticus*	initiation	vancomycin	02:23
6	*vanA/B*-neg *Enterococcus*	*E*. *faecalis*	initiation	ampicillin	02:26
7	*bla*_*KPC*_*-neg S*. *marcescens*	*S*. *marcescens*	initiation	temocillin	02:34
8	*S*. *pneumoniae*	*S*. *pneumoniae*	initiation	penicillin	02:46
9	*mecA*-neg *S*. *aureus*	*S*. *aureus*	initiation	flucloxacillin	03:03
10	*mecA*-neg *S*. *aureus*	*S*. *aureus*	initiation	flucloxacillin	03:37
11	*bla*_*KPC*_-neg *E*. *coli*	*E*. *coli*	initiation	cefuroxime	03:42
12	*mecA*-pos *S*. *aureus*	*S*. *aureus*	broadening	vancomycin	03:47
13	*C*. *albicans*	*C*. *albicans*	initiation	fluconazole	03:50
14	*mecA*-pos *S*. *aureus*	*S*. *aureus*	initiation	vancomycin	03:57
15	*Streptococcus*	*S*. *milleri* group	de-escalation	ampicillin	04:25
16	*S*. *thermophilus*	*S*. *viridans*	initiation	ampicillin	04:33
17	*mecA*-neg *S*. *aureus*	*S*. *aureus*	de-escalation	flucloxacillin	05:27
18	*mecA*-neg *S*. *aureus*	*S*. *aureus*	de-escalation	flucloxacillin	06:33
19	*mecA*-neg *S*. *aureus*	*S*. *aureus*	initiation	flucloxacillin	06:40
20	*bla*_*KPC*_-neg *P*. *aeruginosa*	*P*. *aeruginosa*	broadening	ceftazidime	06:50
21	*mecA*-neg *S*. *aureus*	*S*. *aureus*	initiation	flucloxacillin	07:13
22	*C*. *glabrata*	*C*. *glabrata*	broadening	anidulafungin	08:00
23	*mecA*-neg *S*. *aureus*	*S*. *aureus*	de-escalation	flucloxacillin	11:04
24	bla_*KPC*_-neg *E*. *coli*	*E*. *coli*	de-escalation	cefuroxime	11:40
25	*bla*_*KPC*_-neg *S*. *marcescens*	*S*. *marcescens*	initiation	piperacillin + tazobactam	13:29
26	*L*. *monocytogenes*	*L*. *monocytogenes*	de-escalation	ampicillin	15:52
27	*C*. *glabrata + mecA*-neg *S*. *aureus*	*C*. *glabrata*	initiation	anidulafungin + flucloxacillin	15:53
28	*bla*_*KPC*_-neg *E*. *cloacae* complex	*E*. *cloacae* complex	initiation	temocillin	26:17
29	bla_*KPC*_-neg *E*. *coli*	*E*. *coli*	initiation	ceftriaxone	30:30
30	bla_*KPC*_-neg *E*. *coli*	*E*. *coli*	initiation	cefuroxime	33:55
31	*bla*_*KPC*_-neg *E*. *coli + vanA/B*-neg *Enterococcus*	*E*. *coli + E*. *faecalis*	initiation	cefuroxime + vancoymcin	34:33
32	bla_*KPC*_-neg *E*. *coli*	*E*. *coli* + *C*. *perfringens*	initiation	cefuroxime	37:30
33	*mecA*-neg *Staphylococcus* + *vanA/B*-neg *Enterococcus*	*S*. *epidermidis + E*. *faecalis*	broadening	vancomycin	40:12
34	*bla*_*KPC*_-neg *P*. *aeruginosa*	*P*. *aeruginosa*	broadening	ceftazidime	65:30
35	*bla*_*KPC*_-neg *A*. *baumannii* + *mecA*-neg *Staphylococcus*	*A*. *baumannii* + *S*. *haemolyticus*	broadening	meropenem	108:48

In episode 1–26, the modified treatment upon FA-BCID result was the OAT. In episode 27–35, further tailoring was necessary following ID and AST results. The TAT to OAT is reported in hours:minutes.

Abbreviations: AST, antimicrobial susceptibility testing; FA-BCID, FilmArray blood culture identification; ID, identification; OAT, optimal antimicrobial treatment; TAT, turn-around-time; P1, intervention period.

## Discussion

In this prospective study on critically ill with BSI, FA-BCID testing led to a substantial 10h02 time-reduction to administration of OAT. This beneficial outcome was certainly a combined impact of the FA-BCID test and its around-the-clock realization covering time frames in which culture testing on positive blood bottles was limited or not realized. Thus 80% of the FA-BCID tests enabling a treatment switch occurred outside business hours and 54.3% overnight. This real-time approach has its usefulness for 24h/24 monitored critically ill but could lack clinical responsiveness overnight in other non-critical hospital units.

FA-BCID results improved therapeutic management of 31/110 patients including 26 where the test led to the start-up of the OAT. Principal treatment tailoring consisted in the introduction of an antimicrobial treatment followed by de-escalation and ultimately 3 cases benefitted from antibiotic spectrum broadening. Our restrictive ICU antimicrobial policy limiting antibiotic treatment to patients with a high suspicion of sepsis possibly justifies this distinct observation. Other studies pointed out treatment de-escalation as main consequence of rapid molecular testing [3,5,6]. Even though the inclusion criterion in all studies was identically consisting in the detection of a positive blood culture, few or no information was known on the sepsis status and the empirical treatment of the included patients.

Considering 150 FA-BCID tests were performed in P1, 4,3 tests were required to have 1 test leading to an improved therapeutic outcome. A similar calculation on the subgroup of tests exclusively performed outside working hours concluded only 3,7 tests were necessary to allow a therapeutic tailoring. Prior Gram stain suggesting contamination could similarly limit unnecessary FA-BCID testing.

With regards to microbiological performances, FA-BCID testing identified 96.2% of all on-panel strains covering 85.2% of all microorganisms retrieved through routine culture. Similar results were reported evaluating the FA-BCID panel performances on 161 positive blood culture bottles and calculating the tool’s sensitivity at 99% and the coverage of routine-identified microorganisms at 88.1% [14]. Clinically important off-panel strains missed in our study were mainly anaerobes. Other non-identified strains were Gram-positive bacteria ultimately considered as contaminants and thus of little or no interest. With regards to gene resistance detection, we were able to confirm both optimal sensitivity and specificity for the *mecA* gene but only 100% specificity could be determined for *vanA/B* and *bla*_*KPC*_ genes. Other studies confirmed sensitivity and specificity values > 95% for all 3 resistance genes [14,15]. The low prevalence of *bla*_*KPC*_-positive Gram-negative strains and *vanA/B*-positive *Enterococci* is a common trend in North and Western Europe at the present time [16]. Conversely, in the study of Pardo *et al*. performed in Florida, 46% of all *Enterococcus* BSIs were vancomycin-resistant and FA-BCID results allowed a 16-h gain on the initiation of active therapy compared to routine testing [5].

Molecular blood culture testing has been valorised for it’s ease of use and short time to results. However, this time lapse rarely reflects time to identification results as it depends on how the test is integrated in the laboratory workflow scheme. In our study with FA-BCID testing being performed 24/7, median time to results was reduced with 13h06 compared to MALDI-TOF MS identification in P0. Banerjee *et al*. calculated a time-reduction of 20h42 with 24/7 FA-BCID testing compared to subculture MALDI-TOF MS identification [3]. We believe our routine approach including speeded-up MALDI-TOF MS on young subcultures and blood, might have limited the time impact on identification with the FA-BCID test.

A limitation of our study was the lack of evaluation of the impact of FA-BCID results on length of stay and hospital-costs. Other publications showed main savings were mostly linked to rapid suppression of empirical treatment in contaminations subsequently leading to reduced costs in antibiotic use and reduced costs due to shorter length of stay [5,7,17].

Eventually FA-BCID testing allowed a significant reduction in time to identification and administration of OAT. We hereby consider it as a beneficial add-on identification tool for the diagnosis of BSI in critically ill. However complementary investigations are necessary in settings with high proportions of multi-drug resistant bacteria assuming FA-BCID testing could contribute to speeded-up antimicrobial spectrum reduction and antimicrobial sparing over time.

## Supporting information

S1 File Trend statement Checklist FA-BCID clincal study. (PDF)

S2 File Trial study protocol FA-BCID clinical study_Francais. (PDF)

S3 File Trial study protocol FA-BCID clinical study_English. (PDF)
