# Supplementary material for: The impact of a rapid molecular identification test on positive blood cultures from critically ill with bacteremia: A pre-post intervention study
Source: PLoS One. 2019 Sep 26;14(9):e0223122. doi: 10.1371/journal.pone.0223122 (PMC6762135; doi:10.1371/journal.pone.0223122)
Supplement: S2 File — (PDF) [file pone.0223122.s002.pdf]

Investigateur principal Saint-Luc :

Dr. Alexia VERROKEN, PhD
Microbiologie – Biologie Clinique
Cliniques universitaires St-Luc
02.764.67.66
alexia.verroken@uclouvain.be

Co-investigateur Saint-Luc :

Pr. Pierre-François LATERRE
Soins Intensifs
Cliniques universitaires St-Luc
02.764.27.35
pierre-françois.laterre@uclouvain.be

PROTOCOLE du projet

Clinical impact of the FilmArray® blood culture identification panel performed on positive blood cultures from intensive care patients

Hypothèse

L'utilisation de l'outil moléculaire FilmArray pour l'identification (et la détection de résistances) des hémocultures positives des patients hospitalisés aux soins intensifs accélère significativement le diagnostic microbiologique de la septicémie et améliore la prise en charge thérapeutique du patient affecté.

Protocole

Etude non commerciale prospective non interventionnelle divisée en 2 périodes*.

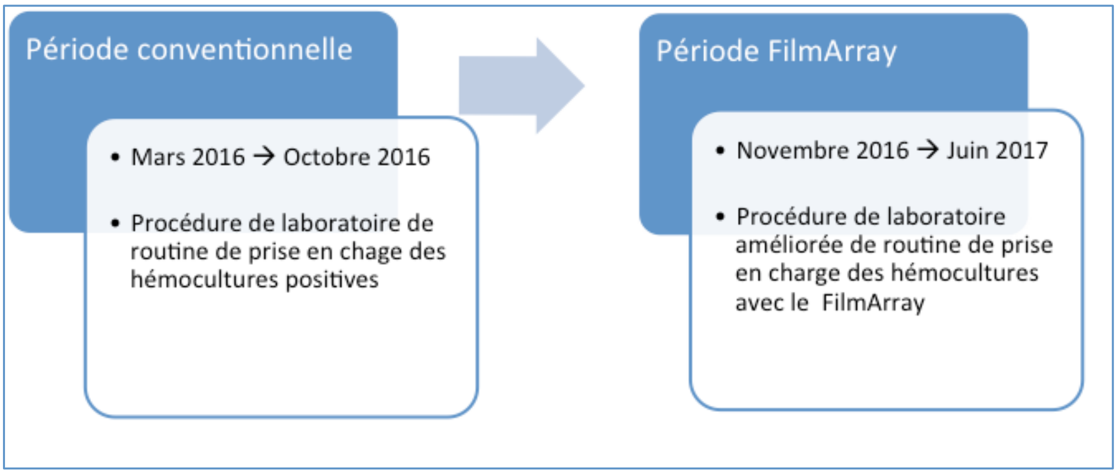

**Qui ?**	Tout patient adulte (>18 ans) avec une hémoculture positive et hospitalisé aux soins intensifs (SIM, SIT, CVI1, CVI2)<br><br>Critères d'exclusion<br>--> Patient qui décède entre le moment où l'hémoculture est prélevée et le moment où l'hémoculture est détectée positive<br>--> Patient bénéficiant exclusivement de soins de confort
**Où ?**	Les analyses de laboratoire sont effectuées au laboratoire de routine de microbiologie des Cliniques universitaires Saint-Luc.
**Quand ?**	Période conventionnelle: mars 2016 → octobre 2016<br>Période FilmArray : octobre 2016 → juin 2017<br><br>La période (en mois) doit correspondre à la récolte de +/- 150 épisodes d'hémocultures positives aux soins intensifs des Cliniques universitaires Saint-Luc.
**Quoi ?**	Selon la période<br><br>- Période conventionnelle : Procédure de laboratoire de routine de prise en charge des hémocultures positives. (Verroken A. et al., PlosOne June 2016)<br><br>- Période FilmArray : Procédure de laboratoire de routine « améliorée » de prise en charge des hémocultures positives incluant le système moléculaire « FilmArray » et la réalisation des analyses 24h/24 et 7j/7.

*Cette étude est non commerciale et réalisée à l'initiative du Dr. VERROKEN Alexia, chef de laboratoire adjoint au laboratoire de microbiologie des Cliniques universitaires Saint-Luc.

Nous estimons que l'étude est prospective car nous collectons les données de façon longitudinale en temps réel tout au long des 2 périodes de l'étude.

Nous considérons cette étude comme non interventionnelle. En effet nous ne faisons pas l'essai de nouveaux médicaments ou de traitement expérimentaux.
La différence entre les 2 périodes siège uniquement dans le temps de rendu des résultats de laboratoire. Dans la période « FilmArray » les résultats d'identification et de détection de résistance sont transmis plus rapidement conduisant à une prescription accélérée de l'antibiotique optimal au patient intensif souffrant d'une septicémie.

**Données patients collectées**	
--> Caractéristiques patients	Age
	Sexe
	Score APACHE II
	Score SOFA
--> Comorbidités	Diabète
	Cancer solide-hématologique
	Pathologie cardiovasculaire
	Pathologie respiratoire chronique
	Pathologie hépatique
	Greffe d'organe
	Hypertension
	Neutrophilie < 500/ µl
	Traitement immunosuppresseur
--> Données microbiologiques	Type bactériémie (septicémie vs contamination)
	Origine infection
	Pathogène(s) identifié(s)
	Résistances détectées (MRSA, VRE, BLSE, CPE)
--> Temps hémocultures positives	Temps jusqu'à l'identification bactérienne
	Méthode d'identification
	Temps jusqu'à la détection partielle de résistance
	Méthode de détection partielle de résistance
	Temps jusqu'à l'antibiogramme complet
--> Outcomes cliniques	Temps jusqu'au traitement antibiotique ciblé
	Changement de traitement post résultat FilmArray
	Type de changement
	Durée de séjour aux soins intensifs
	Durée de séjour à l'hôpital
	Mortalité brute à 30 jours
	Couts prise en charge septicémie

L'ensemble des temps sont calculés à partir du moment où l'hémoculture est détectée positive.

Les données collectées seront d'emblée anonymisées. Ensuite les données obtenus des patients inclus pendant la période « conventionnelle » et les patients inclus pendant la période « FilmArray » seront comparées entre elles et la plus-value clinique de la technique FilmArray sera calculé par méthodes statistiques.
