# Supplementary material for: The impact of a rapid molecular identification test on positive blood cultures from critically ill with bacteremia: A pre-post intervention study
Source: PLoS One. 2019 Sep 26;14(9):e0223122. doi: 10.1371/journal.pone.0223122 (PMC6762135; doi:10.1371/journal.pone.0223122)
Supplement: S3 File — (PDF) [file pone.0223122.s003.pdf]

Principal investigator:

Alexia VERROKEN, MD, PhD
Department of Microbiology
Cliniques universitaires Saint-Luc
02.764.67.66
alexia.verroken@uclouvain.be

Co-investigator Saint-Luc :

Pierre-François LATERRE, MD, PhD
Intensive care Department
Cliniques universitaires Saint-Luc
02.764.27.35
pierre-françois.laterre@uclouvain.be

TRIAL STUDY PROTOCOL

Clinical impact of the FilmArray® blood culture identification panel performed on positive blood cultures from intensive care patients

Hypothesis

The integration of the FilmArray® blood culture identification (and resistance detection) panel on positive blood cultures from intensive care patients speeds up the diagnosis of bacteremia and improves antibiotic management.

Protocol

Prospective non-commercial non-interventional study divided into 2 study periods.

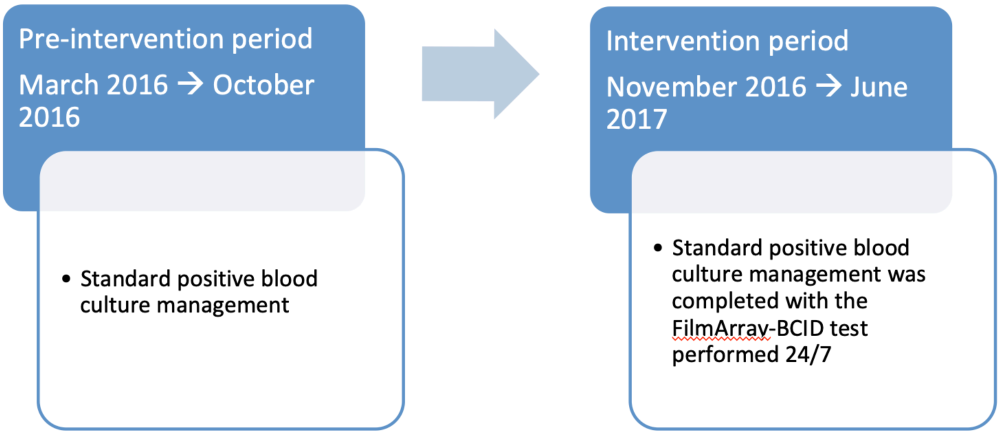

**Who?**	All adult patient (>18 years) staying at the intensive care unit at the time of positive blood culture detection.<br><br>Exclusion criteria<br>→ Patients dying between blood culture sampling and positive detection<br>→ Patients receiving palliative care
**Where?**	Testing is performed at the routine Microbiology Laboratory of the Cliniques universitaires Saint Luc.
**When?**	Expected pre-intervention period: March 2016 → October 2016<br>Expected intervention period: November 2016 → June 2017<br><br>Each period (in months) has to include approximately 160 positive blood culture episodes at the Intensive care Department of the Cliniques universitaires Saint-Luc.
**What?**	According to the period:<br><br>- Pre-intervention period: Routine laboratory procedure for positive blood cultures. (Verroken A. et al., PlosOne June 2016)<br><br>- Intervention period: Routine laboratory procedure for positive blood cultures “improved” by the addition of the FA-BCID test performed 24h/24 and 7d/7.

- <u>non-commercial</u> study initiated by VERROKEN Alexia, chief associate of the Microbiology Department of the Cliniques universitaires Saint Luc.

- <u>Prospective</u> study collecting data in real time along the 2 study periods.

- <u>Non-interventional</u> study as no experimental testing is performed. The difference between the 2 observed periods is the routine management of the positive blood cultures as FilmArray® blood culture identification (FA-BCID) testing is added in the intervention period versus the pre-intervention period.
Time to identification results is the main difference between the two periods. Subsequently this study will measure the impact on improved antibiotic management due to speeded-up laboratory results in the intervention period.

Collected patient data	
--> Patient characteristics	Age
	Sex
	APACHE II score
	SOFA score
--> Comorbidities	Diabetes
	Active solid/hematological malignancy
	Cardiovascular disorder
	Chronic lung disease
	Liver disease
	Solid organ transplant
	Hypertension
	Neutrophil count < 500/µl
	Immunosuppressive treatment
--> Microbiological data	Type of positive blood culture episode (bacteremia versus contamination)
	Source
	Pathogen
	Detected resistances (MRSA, VRE, ESBL, CPE)
--> Positive blood cultures	Time to identification
	Identification method
	Time to result resistance detection test
	Resistance detection test (beta-lacta test, PLP2a test, FA-BCID test)
	Time to complete susceptibility results
--> Clinical outcomes	Time to optimal treatment
	Treatment switch following FA-BCID result
	Type of treatment switch
	Length of stay at the ICU
	Length of stay at the hospital
	30-day mortality
	Cost linked to the bacteremia

All time measurements start at the time the blood culture bottle is detected positive by the incubators.

Collected data are immediately anonymized. Subsequently data of the pre-interventional and interventional period will be compared statistically to evaluate the impact of the FA-BCID test.
